# Low plasma neurofilament light levels associated with raised cortical microglial activation suggest inflammation acts to protect prodromal Alzheimer’s disease

**DOI:** 10.1186/s13195-019-0574-0

**Published:** 2020-01-02

**Authors:** Peter Parbo, Lasse Stensvig Madsen, Rola Ismail, Henrik Zetterberg, Kaj Blennow, Simon F. Eskildsen, Thomas Vorup-Jensen, David J. Brooks

**Affiliations:** 1 Department of Nuclear Medicine and PET Centre, Aarhus University Hospital, Aarhus, Denmark; 2 Department of Clinical Physiology, Viborg Regional Hospital, Viborg, Denmark; 3 Department of Psychiatry and Neurochemistry, Institute of Neuroscience and Physiology, The Sahlgrenska Academy at the University of Gothenburg, Mölndal, Sweden; 4 Clinical Neurochemistry Laboratory, Sahlgrenska University Hospital, Mölndal, Sweden; 5 Department of Neurodegenerative Disease, UCL Queen Square Institute of Neurology, London, UK; 6 UK Dementia Research Institute at UCL, London, UK; 7 Center of Functionally Integrative Neuroscience, Aarhus University, Aarhus, Denmark; 8 Department of Biomedicine/Interdisciplinary Nanoscience Centre (iNANO), Aarhus University, Aarhus, Denmark; 9 Institute of Neuroscience, University of Newcastle upon Tyne, Newcastle upon Tyne, UK

**Keywords:** Neurofilament light, Microglia, Inflammation, PET, PK11195, MCI, Alzheimer

## Abstract

**Background:**

Plasma and cerebrospinal fluid levels of neurofilament light (NfL), a marker of axonal degeneration, have previously been reported to be raised in patients with clinically diagnosed Alzheimer’s disease (AD). Activated microglia, an intrinsic inflammatory response to brain lesions, are also known to be present in a majority of Alzheimer or mild cognitive impaired (MCI) subjects with raised β-amyloid load on their positron emission tomography (PET) imaging. It is now considered that the earliest phase of inflammation may be protective to the brain, removing amyloid plaques and remodelling synapses. Our aim was to determine whether the cortical inflammation/microglial activation load, measured with the translocator protein marker ^11^C-PK11195 PET, was correlated with plasma NfL levels in prodromal and early Alzheimer subjects.

**Methods:**

Twenty-seven MCI or early AD cases with raised cortical β-amyloid load had ^11^C-*(R)*-PK11195 PET, structural and diffusion magnetic resonance imaging, and levels of their plasma NfL measured. Correlation analyses were performed using surface-based cortical statistics.

**Results:**

Statistical maps localised areas in MCI cases where levels of brain inflammation correlated inversely with plasma NfL levels. These areas were localised in the frontal, parietal, precuneus, occipital, and sensorimotor cortices. Brain inflammation correlated negatively with mean diffusivity (MD) of water with regions overlapping.

**Conclusion:**

We conclude that an inverse correlation between levels of inflammation in cortical areas and plasma NfL levels indicates that microglial activation may initially be protective to axons in AD. This is supported by the finding of an inverse association between cortical water diffusivity and microglial activation in the same regions. Our findings suggest a rationale for stimulating microglial activity in early and prodromal Alzheimer cases—possibly using immunotherapy. Plasma NfL levels could be used as a measure of the protective efficacy of immune stimulation and for monitoring efficacy of putative neuroprotective agents.

**Electronic supplementary material:**

The online version of this article (10.1186/s13195-019-0574-0) contains supplementary material, which is available to authorized users.

## Background

Neurofilaments are found in the cytoplasm of neurons and act to transport nutrients and other molecules along axons. The subunit proteins constituting neurofilaments include neurofilament light (NfL), medium and heavy (L, M and H) proteins based on their molecular weight [1]. The neurofilaments control axonal diameter, and it has been reported that cerebrospinal fluid (CSF) levels of NfL provide a non-specific marker of axonal degeneration. Patients with amyotrophic lateral sclerosis where pyramidal tract involvement is present show greatly raised CSF NfL levels, while those with only peripheral motor neuron disease (progressive muscular atrophy) show smaller elevations [2, 3]. Multiple sclerosis cases in the chronic progressive phase of their disease also show raised CSF NfL, but those who have early relapsing remitting disease show only modest elevations [4]. Raised levels of CSF NfL have also been reported in Alzheimer’s disease (AD) and Huntington’s disease [2, 5, 6]. No relationship was found between cognitive status and CSF NfL levels in AD patients, though disability of Huntington’s disease cases, when rated with the UHDRS, correlated with CSF NfL levels [6].

More recently, an ultrasensitive digital array for enzyme-linked immunosorbent assay (ELISA) of proteins including NfL has been developed [7], which allows reliable detection of single molecules of NfL in plasma or serum. NfL plasma levels were found to correlate strongly with those measured in CSF. A large number of studies have now shown that blood NfL is a robust biomarker of neuronal injury, irrespective of the cause [8]. In the context of AD, serum NfL has been found to mark the onset of neurodegeneration in subjects at risk for familial disease [9]. A recent study found increased plasma NfL in AD patients compared to nondemented controls [10]. A larger study involving the Alzheimer’s Disease Neuroimaging Initiative (ADNI-1) cohort reported raised plasma NfL in sporadic mild cognitive impairment (MCI) and Alzheimer cases [11]. The plasma NfL levels correlated with reduced CSF β-amyloid 1–42 and raised CSF tau in the MCI cohort. At baseline, high plasma NfL levels in the MCI and AD cohort were associated with worse Mini-Mental State Examination (MMSE) and ADAS-COG 11 scores and subjects had larger ventricular and smaller hippocampal volumes and thinner cortices.

Microglia are part of the intrinsic immune defence of the brain but normally exist in a resting state monitoring the local constituents of brain extracellular fluid [12]. In AD, there is abnormal formation of extracellular plaques of β-amyloid protein fibrils and intraneuronal tangles of hyperphosphorylated tau. This characteristic pathology of AD is associated with an inflammatory response which involves activated microglia surrounding plaques and degenerating neurons [13]. Activated, but not resting, microglia express the translocator protein (TSPO) on the outer membranes of their mitochondria [14]. ^11^C-PK11195 positron emission tomography (PET) is an in vivo marker of the TSPO expressed by activated microglia. Recently, we reported that 80% of β-amyloid-positive MCI cases showed associated active inflammation and raised TSPO [15]. However, whether the inflammation was playing a protective or cidal role in the disease was unclear. It has been reported in established AD that levels of temporoparietal cortical microglial activation correlate inversely with cognitive ratings on the MMSE [16]. This suggests that the microglial phenotype may be toxic in these subjects. However, microglia are known to also express protective phenotypes releasing growth factors, clearing amyloid fibrils, and remodelling damaged synapses. Here, we report the presence of an inverse association of plasma NfL levels, acquired from cases with either prodromal (β-amyloid-positive MCI) or early clinical β-amyloid-positive AD, with their levels of cortical microglial activation measured with ^11^C-*(R)*-PK11195 PET [17]. These findings are corroborated by measurements of atrophy and microstructural integrity using magnetic resonance imaging (MRI).

## Methods

### Study subjects

Subjects with MCI and clinical AD were recruited from memory clinics and by advertisement as part of an ongoing longitudinal PET study and were assessed as previously described [15]. In this report, we included amyloid-positive MCI/AD subjects who had measures of plasma NfL, an ^11^C-PK11195 PET scan, and whole-brain MRI with structural and diffusion-weighted imaging (DWI). The MCI subjects fulfilled the Petersen clinical criteria while the AD patients were diagnosed by dementia specialists using ICD-10 criteria. All subjects were assessed with MMSE and Clinical Dementia Rating (CDR). The project was approved by the regional ethics committee and all subjects gave their written informed consent.

### Plasma NfL levels

Plasma NfL concentrations were measured using an ultrasensitive in house ‘single molecule array’ (SIMOA) ELISA assay, as previously described in detail [7]. The measurements were performed by a board-certified laboratory technician using a single batch of reagents.

### Brain imaging

MRI was performed on a Skyra 3 Tesla system (Siemens, Erlangen, Germany). An MP2RAGE (Magnetization Prepared Rapid Gradient-Echo with two gradient echo images) sequence was used for co-registration of MRI with PET, normalisation into standard space, generation of grey matter (GM) masks, and measurements of cortical thickness. DWI was obtained using a fast, clinically practical, diffusion kurtosis imaging sequence [18, 19]. Acquisitions included one b = 0 image, three b = 1000 s/mm^2^, and nine b = 2500 s/mm^2^, all acquired with 2.3-mm isotropic voxels, FOV = 220 × 220 mm^2^ in 38 slices, TR = 12.4 s, TE = 0.107 s, and TI = 2.1 s. Mean diffusivity (MD) was calculated from the DWI sequence. MD is an imaging biomarker of early axonal degenerative changes in AD [20].

Subjects were scanned using a High Resolution Research Tomograph (ECAT HRRT; CTI/Siemens, Knoxville, TN) as previously described [15, 21]. A mean 392 MBq dose of ^11^C-*(R)*-(1-[2-chlorophenyl]-*N*-methyl-*N*-[1-methyl-propyl]-3-isoquinolinecarboxamide) (abbreviated PK11195) was injected intravenously. The total dynamic scan time was 60.5 min (list mode 3D acquisition).

PET images were reconstructed with a 3D-OSEM (ordered subset expectation maximum) with 10 iterations and 16 subsets. Point-spread function (PSF) reconstruction was applied to minimise partial volume effects, improve image quality, contrast and quantitative accuracy, and achieve a reconstructed resolution of 2.5 mm. Images were not partial volume corrected.

### Image analyses using cortical surface maps

Parametric maps of binding potential (BP_ND_) were generated at a voxel level from the dynamic ^11^C-*(R)*-PK11195 PET images using the Simplified Reference Tissue Model (SRTM) [22] implemented in Matlab. As all anatomical regions in the brain can potentially show specific PK11195 binding in Alzheimer’s disease due to local pathology or downstream disconnection, a Supervised Cluster Analysis with 6 classes [23] (SVCA6) was used to localise a distributed cluster of voxels from the dynamic images of each MCI/AD case, which provided a reference tissue input function representing normal GM kinetics.

Surface-based cortical statistics were used to perform the analyses. For each subject, the cortical surface was extracted from the individual MP2RAGE image using Fast Accurate Cortex Extraction (FACE) [24]. To map the PK11195 PET and DWI signal onto the surface, individual cortical surfaces were transformed into the PET/DWI native space, respectively, using a rigid body co-registration with the MP2RAGE image. The signal was then interpolated and mapped to the surface approximating the middle cortical layer, in order to minimise the influence of partial volume effects. Before mapping the PK11195 signal, the PET image was smoothed using a 4-mm full width at half maximum (FWHM) Gaussian filter to compensate for potential inaccuracies in the co-registration. Individual surfaces were registered to the cortical surface of an average non-linear anatomical template in the MNI space using a feature-driven surface registration algorithm [25, 26]. Parameter values were then mapped to the average surface and smoothed using a 20-mm FWHM geodesic Gaussian kernel. Smoothing along the cortical surface eliminates unwanted blurring across gyri caused by smoothing in voxel space.

Cortical thickness was determined by estimating the distance between the white matter (WM)-GM surface and the GM-CSF surface at each location of the cortical surface, producing a cortical thickness surface map for each individual. Using the same approach as before, individual cortical thickness maps were mapped to the average surface and smoothed.

### Statistical analyses

Correlation between parameters was calculated at each point/vertex on the cortical surface using a linear regression model implemented in Python 3.7.3 (Python Software Foundation). This produced a statistical map of the cortical surface with each vertex indicating regression slopes different from zero. Vertices with positive *t*-values indicate positive correlations and vice versa. In order to adjust for multiple comparisons, results from both positive and negative correlations were family-wise error rate (FWER) corrected using cluster-extent-based thresholding with varying primary cluster-defining thresholds for significance of *p* < 0.01 and *p* < 0.001 (both shown as overlay in figures). We chose to show thresholds of *p* < 0.01 and *p* < 0.001 as our primary findings. In Additional files 1, 2, 3, and 4, we have added the more lenient threshold of *p* < 0.05 to better comprehend the patterns of correlations. The cluster-extent threshold for significance was calculated using a Gaussian random field implemented in Statistical Parametric Mapping 12 (SPM12; Wellcome Trust Centre for Neuroimaging) taking into account the estimated intrinsic smoothness based in residual maps [27]. Visbrain was used for visualisation of the surface-based statistical maps [28]. Spearman’s Rho (ρ) was applied to test for correlation between NfL–MMSE and NfL–CDR sum-of-boxes (CDR-SOB).

## Results

Twenty-seven MCI/AD subjects (8 females/19 males) were included in the analyses. The subjects had a mean age of 73.6 years (± 6.1), mean MMSE score of 25.9 (± 3.0), and a mean plasma NfL level of 27.9 pg/mL (± 10.7). One AD subject had no DWI sequence due to claustrophobia, so MD analyses only include 26 subjects.

**Fig. 1:**
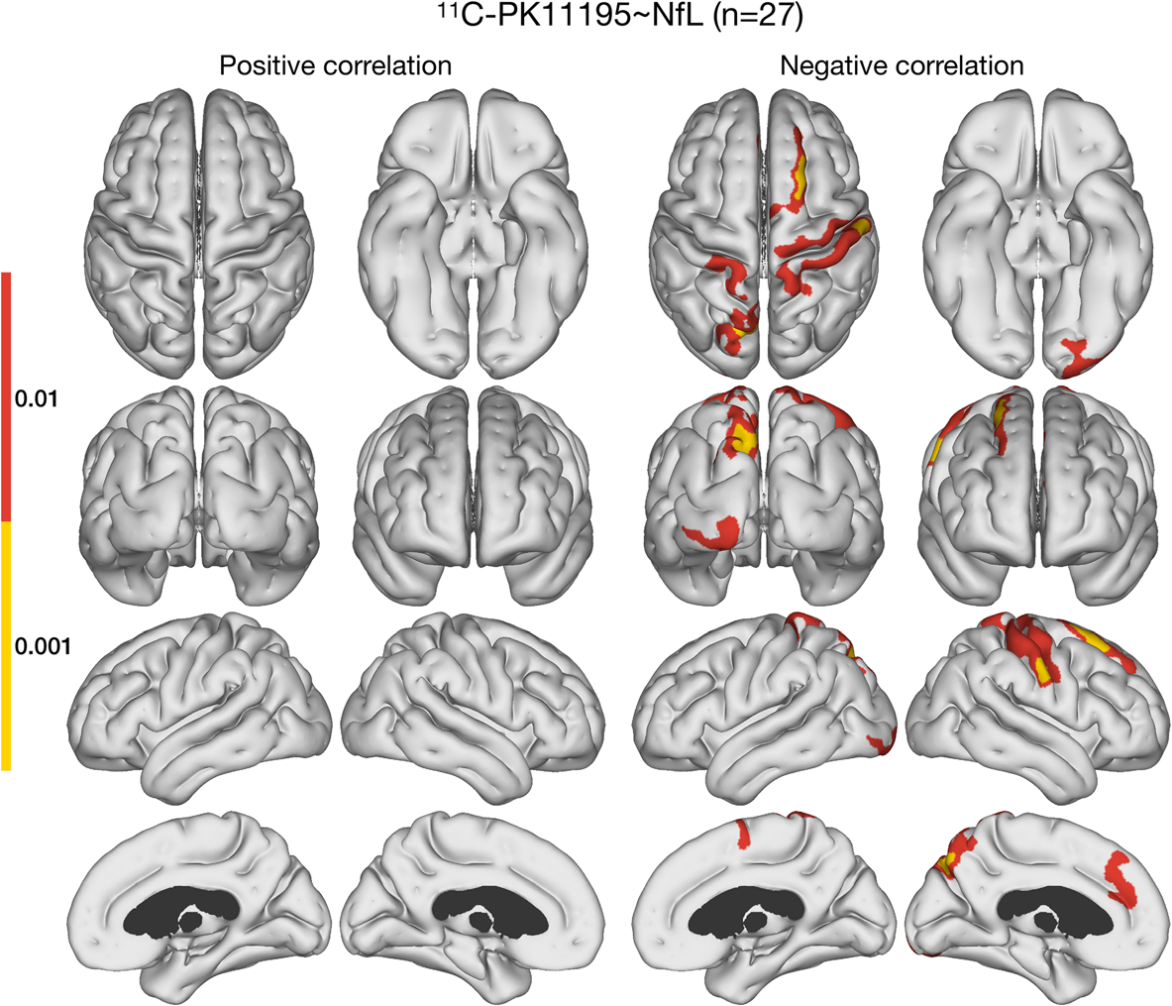
Correlations between ^11^C-PK11195 BP_ND_ and plasma NfL. Surface-based cortical statistics displaying negative correlations between ^11^C-PK11195 BP_ND_ and plasma NfL levels in frontal, parietal, precuneus, occipital, and sensorimotor areas across 27 prodromal and early AD cases. The presented maps are cluster-level FWER corrected at *p* < 0.05, with two different cluster-defining primary thresholds: *p* < 0.01 and *p* < 0.001

**Fig. 2:**
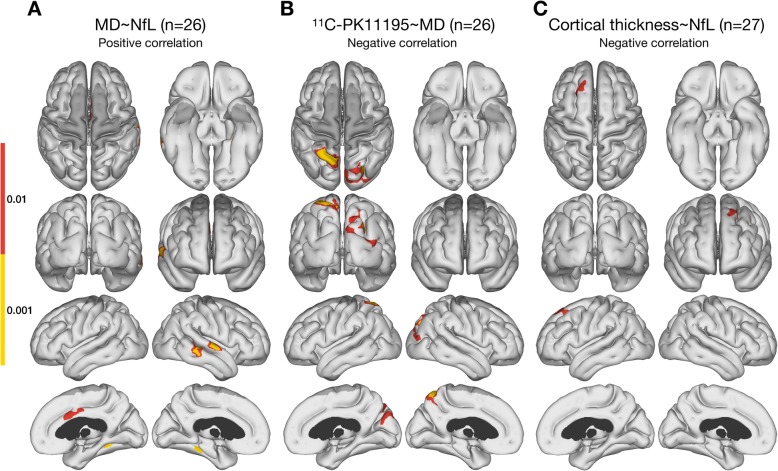
Surface based cortical statistics. **a** Areas with positive correlation between MD and levels of plasma NfL in temporal and cingulate regions. **b** Areas with negative correlation between MD and ^11^C-PK11195 BP_ND_ in parietal, precuneus, and occipital regions. **c** Areas with negative correlation between cortical thickness and NfL in frontal region. The presented maps are cluster-level FWER corrected at *p* < 0.05, with two different cluster-defining primary thresholds: *p* < 0.01 and *p* < 0.001. Dark grey indicate areas not covered in all subjects by DWI

Plasma NfL correlated inversely with PK11195 BP_ND_ levels in areas of frontal, parietal, precuneus, occipital, and sensorimotor cortices in the 27 MCI/AD cases (Fig. 1). MD correlated positively with plasma NfL levels in areas of the temporal lobes and the cingulate cortex (Fig. 2a). Levels of MD correlated inversely with PK11195 BP_ND_ primarily in parietal areas (Fig. 2b) and overlapped with areas of PK11195–NfL inverse correlation. It should be noted that not all subjects had whole-brain coverage for the DWI sequence, which led to lack of statistical inference in the most superior and inferior parts of the cerebrum (shown as dark grey areas on the figures involving MD). Finally, cortical thickness correlated inversely with plasma NfL levels in a single cluster in the left frontal lobe (Fig. 2c). There were no correlations between cortical thickness and PK11195 binding. Additionally, no correlations were detected between MMSE ratings and plasma NfL levels (ρ = − 0.057, *P* = 0.78) or cortical PK11195 binding, or between CDR-SOB scores and plasma NfL levels (ρ = 0.257, *P* = 0.20) or PK11195 binding.

## Discussion

This paper is the first to report in vivo an inverse association of plasma NfL concentrations with levels of cortical microglial activation imaged with PET in patients with prodromal or early mild AD confirmed by the presence of raised cortical β-amyloid deposition detected with ^11^C-PiB PET. Since plasma NfL levels are now a well-established biomarker for the extent of axonal damage, these results suggest that cortical microglial activation in early Aβ-positive individuals may initially play a protective role.

Plasma NfL levels have been shown to correlate strongly with those in CSF [7]. A recent study by Ashton et al. found that plasma NfL concentrations correlated with both severity of post-mortem neurofibrillary tangle pathology and nerve cell loss from neurodegeneration [29]. We have previously found PK11195 binding to be associated with amyloid load in prodromal AD [15]. In that series, it was noted that amyloid-positive MCI/AD subjects with absent or very low tau load still presented with high PK11195 binding [21]. This finding emphasises that β-amyloid aggregation can trigger an inflammatory brain response in vivo independent and ahead of the presence of tau tangles.

Activated microglia can be seen surrounding amyloid plaques and degenerating neurons containing tau tangles in Alzheimer brain slices with immunohistochemical stains [30]. The role of these microglia is still being debated. It has been suggested that initially microglia may be protective, attempting to clear toxic amyloid oligomers in early disease and releasing nerve growth factors. Subsequently, they fail to achieve this function and their levels decline. A later wave of microglia is then activated as tau tangles form and their load rises and these microglia express a toxic phenotype releasing cytokines which kill neurons and their axons so driving disease progression [17]. PK11195 PET is unable to discriminate between protective and cytotoxic activated microglia as both these microglial phenotypes express TSPO. If the majority of activated microglia in our MCI and early AD cohort exhibit a protective phenotype, then this could explain our observation of an inverse correlation between cortical microglial load and plasma NfL levels.

Surface-based cortical statistics showed that areas where brain inflammation levels correlated with plasma NfL levels in our MCI/AD group were localised in frontal, cingulate, parietal, precuneus, occipital, and sensorimotor cortices. The first four areas are particularly targeted by amyloid deposition [31], while occipital and primary sensorimotor cortices usually are relatively spared. Pyramidal cells in layers 3 and 5 of association cortical areas project to the brainstem and spinal cord, and hence, it is most likely that degeneration of these axons is responsible for the leakage of NfLs into the plasma.

We also found cortical areas where there was an inverse correlation between MD and PK11195 binding. These areas overlapped with areas of NfL–PK11195 inverse association, but were smaller in extent. As both NfL and MD are considered surrogate markers of axon loss, this result could corroborate a model of activated microglia in areas of intact microstructure. MD correlated positively with NfL in areas associated with early involvement in AD. Specifically, the left medial temporal lobe, the right lateral temporal lobe, and the cingulate cortex showed this association. These areas may represent a later disease stage where microglia are no longer protective and neurodegeneration has started. We found only a few small areas of inverse correlation between plasma NfL levels and cortical thickness. Cortical thickness is not sensitive to the initial neurodegeneration on an individual basis, and with the small sample size studied here; the statistical power is probably insufficient to pick up strong correlations for cortical thickness measurements.

While other studies have found association between cognitive function, e.g. MMSE and NfL [32], we found no associations between cognitive function (MMSE, CDR-SOB) and neither plasma NfL levels or cortical PK11195 binding. This could be due to our small sample size and the fact that it consist of MCI/early AD cases.

### Limitations

Our study was cross-sectional, so we are unable to delineate the longitudinal relationship between plasma NfL and brain inflammation in AD from these data. The current study is single centre and based on a relatively small sample of early AD cases, so the results need to be confirmed in a larger cohort. We chose to visualise cluster-defining thresholds of *p* < 0.01 and *p* < 0.001. In other imaging modalities, a threshold of *p* < 0.01 is considered too lenient [33]; however, this has not been tested for PET and diffusion MR imaging. Compared to newer TSPO tracers now available, PK11195 PET has a low signal-to-noise ratio due to a high background signal. In favour of PK11195 PET, however, is the finding that the signals are not significantly influenced by the TSPO polymorphism expressed by subjects unlike newer PET TSPO markers; this simplifies the interpretation of imaging findings.

## Conclusion

We have found that in early and prodromal Alzheimer patients raised plasma levels of NfL protein, a marker of axonal neurodegeneration, are inversely correlated with raised levels of PK11195 binding, a marker of microglial activation / neuroinflammation, in cortical areas. As raised plasma NfL reflect active neurodegeneration, our finding supports the view that inflammation initially plays a protective role in AD. Both these modalities have the potential to provide useful biomarkers in future studies on the efficacy of neuroprotective agents. The inverse correlation of levels of raised activated microglia in early AD with reduced plasma levels of NfL could suggest that initially boosting microglia activation, possibly with immunotherapy targeting abnormal protein aggregations, in early disease could be protective, but this hypothesis needs to be tested in further studies.

## Additional files

Additional file 1: **Figure S1.** Correlations between ^11^C-PK11195 BP_ND_ and plasma NfL levels at three different cluster defining thresholds: *p* < 0.05, *p* < 0.01, *p* < 0.001. All are cluster-level FWER corrected at *p* < 0.05. Including scatter plot of subject mean values of the largest cluster surviving *p* < 0.05 FWER correction with a primary cluster defining threshold of *p* < 0.01 (indicated by black arrow).

Additional file 2: **Figure S2.** Correlations between MD and plasma NfL levels at three different cluster defining thresholds: *p* < 0.05, *p* < 0.01, *p* < 0.001. All are cluster-level FWER corrected at *p* < 0.05. Including scatter plot of subject mean values of the largest cluster surviving *p* < 0.05 FWER correction with a primary cluster defining threshold of *p* < 0.01 (indicated by black arrow). Dark grey indicate areas not covered in all subjects by DWI.

Additional file 3: **Figure S3.** Correlations between ^11^C-PK11195 BP_ND_ and MD at three different cluster defining thresholds: *p* < 0.05, *p* < 0.01, *p* < 0.001. All are cluster-level FWER corrected at *p* < 0.05. Including scatter plot of subject mean values of the largest cluster surviving *p* < 0.05 FWER correction with a primary cluster defining threshold of *p* < 0.01 (indicated by black arrow). Dark grey indicate areas not covered in all subjects by DWI.

Additional file 4: **Figure S4.** Correlations between cortical thickness and plasma NfL levels at three different cluster defining thresholds: *p* < 0.05, *p* < 0.01, *p* < 0.001. All are cluster-level FWER corrected at *p* < 0.05. Including scatter plot of subject mean values of the largest cluster surviving *p* < 0.05 FWER correction with a primary cluster defining threshold of *p* < 0.01 (indicated by black arrow).

## Data Availability

The datasets used and/or analysed during the current study are available on reasonable request.
