# Supplementary figures and images for: Low plasma neurofilament light levels associated with raised cortical microglial activation suggest inflammation acts to protect prodromal Alzheimer’s disease
Source: Alzheimers Res Ther. 2020 Jan 2;12:3. doi: 10.1186/s13195-019-0574-0 (PMC6941285; doi:10.1186/s13195-019-0574-0)

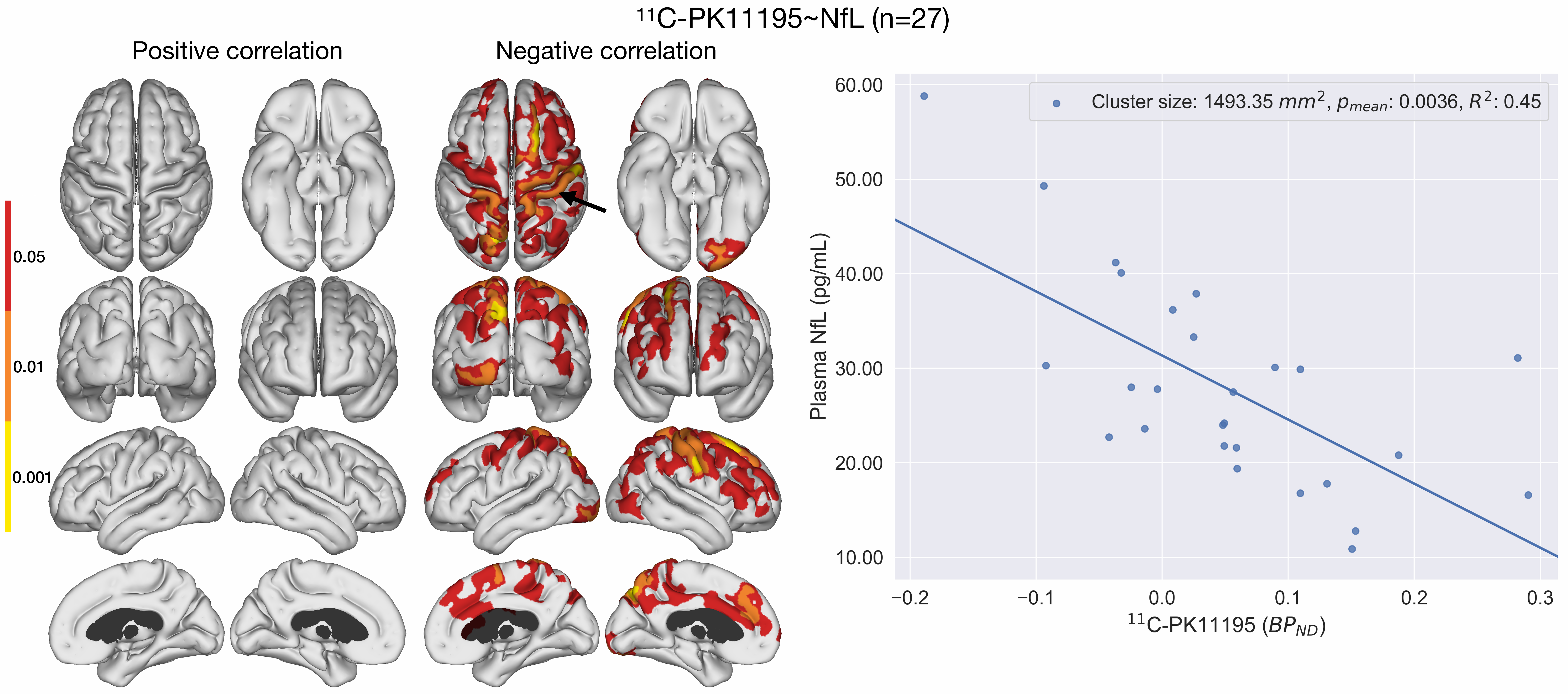

Supplement: Supplementary file 1 — Figure S1. Correlations between 11C-PK11195 BPND and plasma NfL levels at three different cluster defining thresholds: p < 0.05, p < 0.01, p < 0.001. All are cluster-level FWER corrected at p < 0.05. Including scatter plot of subject mean values of the largest cluster surviving p < 0.05 FWER correction with a primary cluster defining threshold of p < 0.01 (indicated by black arrow). [file 13195_2019_574_MOESM1_ESM.tif]

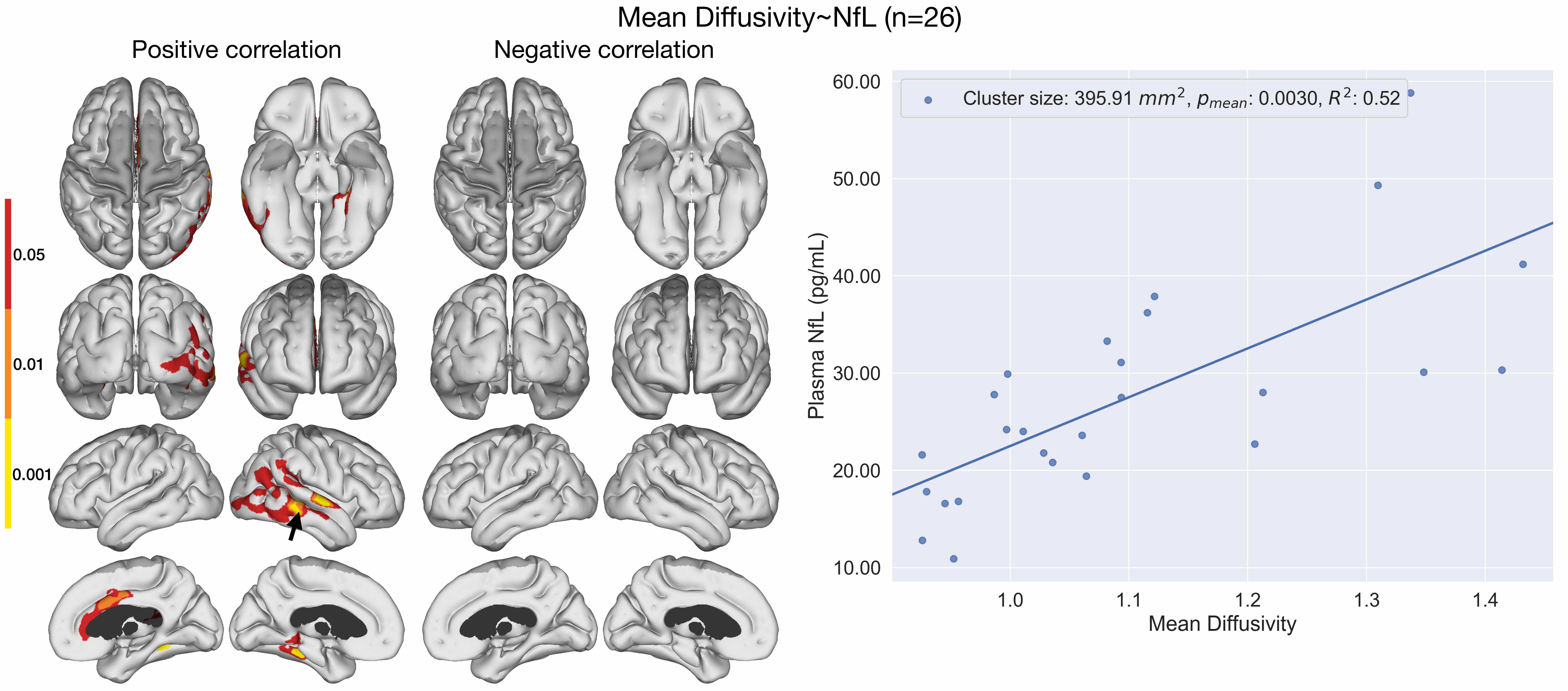

Supplement: Supplementary file 2 — Figure S2. Correlations between MD and plasma NfL levels at three different cluster defining thresholds: p < 0.05, p < 0.01, p < 0.001. All are cluster-level FWER corrected at p < 0.05. Including scatter plot of subject mean values of the largest cluster surviving p < 0.05 FWER correction with a primary cluster defining threshold of p < 0.01 (indicated by black arrow). Dark grey indicate areas not covered in all subjects by DWI. [file 13195_2019_574_MOESM2_ESM.tif]

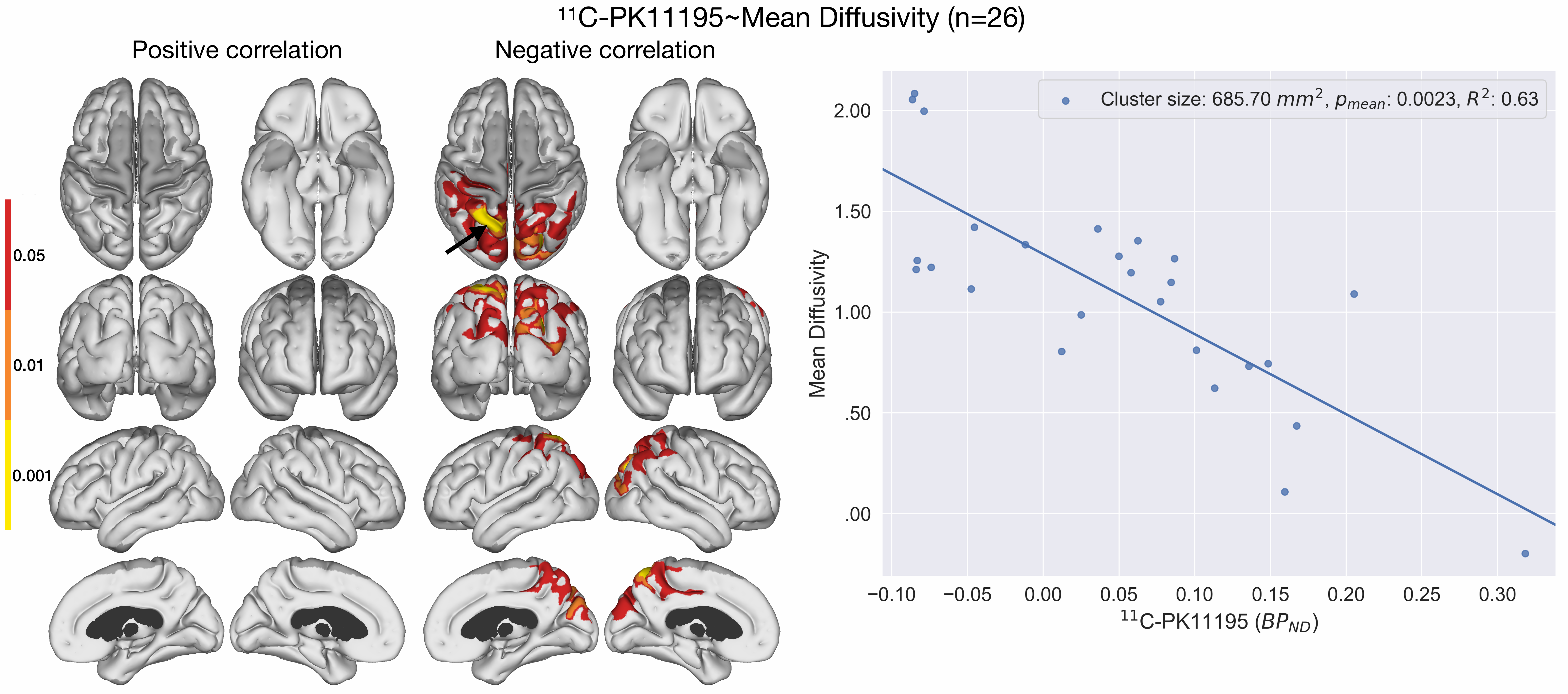

Supplement: Supplementary file 3 — Figure S3. Correlations between 11C-PK11195 BPND and MD at three different cluster defining thresholds: p < 0.05, p < 0.01, p < 0.001. All are cluster-level FWER corrected at p < 0.05. Including scatter plot of subject mean values of the largest cluster surviving p < 0.05 FWER correction with a primary cluster defining threshold of p < 0.01 (indicated by black arrow). Dark grey indicate areas not covered in all subjects by DWI. [file 13195_2019_574_MOESM3_ESM.tif]

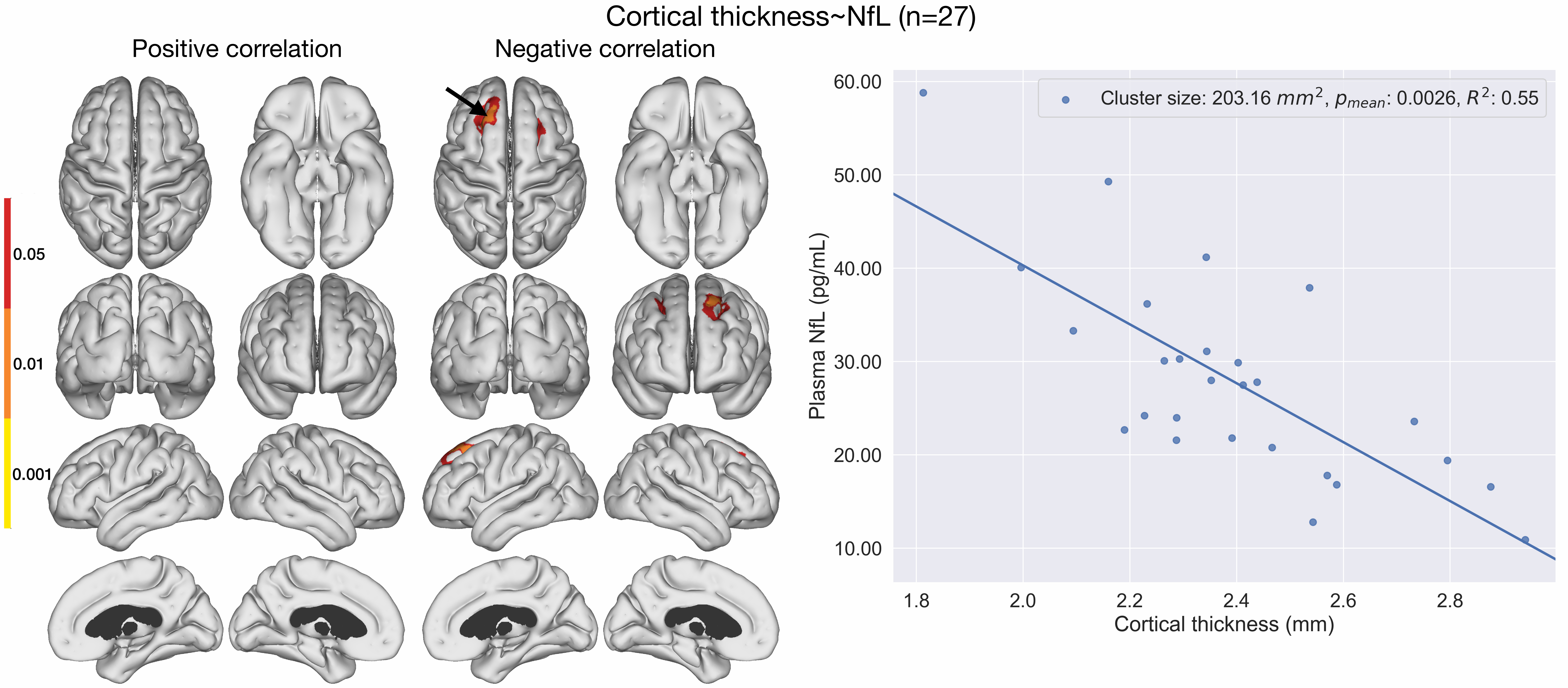

Supplement: Supplementary file 4 — Figure S4. Correlations between cortical thickness and plasma NfL levels at three different cluster defining thresholds: p < 0.05, p < 0.01, p < 0.001. All are cluster-level FWER corrected at p < 0.05. Including scatter plot of subject mean values of the largest cluster surviving p < 0.05 FWER correction with a primary cluster defining threshold of p < 0.01 (indicated by black arrow). [file 13195_2019_574_MOESM4_ESM.tif]
